# MicroRNA‐130a regulates neurological deficit and angiogenesis in rats with ischaemic stroke by targeting XIAP

**DOI:** 10.1111/jcmm.15732

**Published:** 2020-08-13

**Authors:** Wenjing Deng, Chenghe Fan, Yanan Zhao, Yuewei Mao, Jiajia Li, Yonggan Zhang, Junfang Teng

**Affiliations:** ^1^ The Neurology Intensive Care Unit The First Affiliated of Zhengzhou University. Zhengzhou Henan China; ^2^ The Vascular Surgery Department Zhengzhou Central Hospital Affiliated Hospital of Zhengzhou University. Zhengzhou Henan China; ^3^ The Neurology Department Zhengzhou Central Hospital Affiliated Hospital of Zhengzhou University. Zhengzhou Henan China; ^4^ The Vascular Surgery Department The First Affiliated of Zhengzhou University. Zhengzhou Henan China

**Keywords:** angiogenesis, ischaemic stroke, microRNA‐130a, middle cerebral artery occlusion, neurological deficit, X‐linked inhibitor of apoptosis protein

## Abstract

MicroRNAs (miRNAs) have already been proposed to be implicated in the development of ischaemic stroke. We aim to investigate the role of miR‐130a in the neurological deficit and angiogenesis in rats with ischaemic stroke by regulating X‐linked inhibitor of apoptosis protein (XIAP). Middle cerebral artery occlusion (MCAO) models were established by suture‐occluded method, and MCAO rats were then treated with miR‐130a mimics/inhibitors or/and altered XIAP for detection of changes of rats’ neurological function, nerve damage and angiogenesis in MCAO rats. The oxygen‐glucose deprivation (OGD) cellular models were established and respectively treated to determine the roles of miR‐130a and XIAP in neuronal viability and apoptosis. The expression levels of miR‐130a and XIAP in brain tissues of MCAO rats and OGD‐treated neurons were detected. The binding site between miR‐130a and XIAP was verified by luciferase activity assay. MiR‐130a was overexpressed while XIAP was down‐regulated in MCAO rats and OGD‐treated neurons. In animal models, suppressed miR‐130a improved neurological function, alleviated nerve damage and increased new vessels in brain tissues of rats with MCAO. In cellular models, miR‐130a inhibition promoted neuronal viability and suppressed apoptosis. Inhibited XIAP reversed the effect of inhibited miR‐130a in both MCAO rats and OGD‐treated neurons. XIAP was identified as a target of miR‐130a. Our study reveals that miR‐130a regulates neurological deficit and angiogenesis in rats with MCAO by targeting XIAP.

## 1 INTRODUCTION

Ischaemic stroke is a fatal condition (the lack of blood flow to the brain) which is capable of causing neurological deficit and death.^1^, ^2^ It has been proposed that many factors are attributed to this disease, including high blood pressure, lack of exercise, unhealthy lifestyle and diabetes mellitus.^3^ The standard treatment for ischaemic stroke is intravenous recombinant tissue plasminogen activator (t‐PA), but over fifty per cent of treated patients was not fully healed and even dead in the end, and its alternative one endovascular therapy does not exceed it.^4^ Thus, novel targets are urgently needed for the treatment of ischaemic stroke.

MicroRNAs (miRNAs) are a collection of short non‐coding RNA molecules with the length of about 22 nucleotides, which are capable of regulating gene expression by binding to 3’‐untranslated regions (3’‐UTR) of target mRNAs in the cytoplasm.^5^, ^6^, ^7^ A previous study has revealed that miR‑150 polymorphisms may facilitate the emergence of ischaemic stroke and probably serve as biomarkers for ischaemic stroke prediction.^8^ It has been elucidated that down‐regulated miR‐134 increases HSPA12B level to defend neural cells from ischaemic injury in ischaemic stroke.^9^ Interestingly, miR‐130a has been reported to exert neuroprotective effects against ischaemic stroke,^10^ and it has also been verified that miR‐130a regulated cerebral ischaemia‐induced blood‐brain barrier permeability.^11^ X‐linked inhibitor of apoptosis protein (XIAP), an extremely critical member of inhibitor of apoptosis protein (IAP) family, is a cytosolic inhibitor of caspases‐3, −7 and −9.^12^, ^13^ It was confirmed by Petra Obexer et al that XIAP is unconventionally expressed in various cancers, and it plays a significant part in the regulation of death resistance and functions as a therapeutic target for cancer therapy.^14^ XIAP was proposed to be a vital regulator of sex difference in stroke, and miR‐23a can facilitate the differences by regulating XIAP.^15^ Zhang et al have demonstrated that XIAP is targeted by miR‐130 and down‐regulated miR‐130a promotes cisplatin resistance of cells in ovarian cancer by regulating XIAP.^16^ Nevertheless, the function of miR‐130a targeting XIAP in ischaemic stroke has scarcely been investigated.

Experimental focal cerebral ischaemia models are employed to mimic human stroke. Rodent models of focal cerebral ischaemia have been developed using middle cerebral artery occlusion (MCAO).^17^ Moreover, the in vitro cerebral ischaemic model was usually established by OGD exposure in neurons.^18^ Thus, our study is intended for the exploration of miR‐130a's role in MCAO rats and OGD‐treated neurons to clarify its effect on ischaemic stroke by regulating XIAP.

## 2 MATERIALS AND METHODS

### 2.1 Ethics statement

Animal experiments were strictly in line with the Guide to the Management and Use of Laboratory Animals issued by the National Institutes of Health. The protocol of animal experiments was approved by the Institutional Animal Care and Use Committee of The First Affiliated of Zhengzhou University.

### 2.2 MCAO model establishment

Rat models of permanent MCAO were made by Longa's suture‐occluded method.^19^ Healthy male Sprague‐Dawley (SD) rats, weighing 230‐270 g and purchased from Shanghai SLAC Laboratory Animal Co., Ltd. (Shanghai, China), were anaesthetized by intraperitoneal injection of 10% chloral hydrate (0.3 mL/100 g) and placed on the operating table in a supine position, with limbs fixed by strings. An incision was made in the middle of the neck, and common carotid artery (CCA), external carotid artery (ECA) and internal carotid artery (ICA) in the right were separated. A suture and a bulldog clamp were prepared at the proximal end of the ICA and at the distal end, respectively. Then, another incision was made at the CCA, and the prepared suture was made through the incision from the start of middle cerebral artery (MCA) to the proximal end of the anterior cerebral artery (ACA) to a depth of 17 to 19 mm. Next, the suture was fixed and that moment was recorded as the time for the onset of regional cerebral ischaemia. The incision was sutured layer by layer and disinfected, and rats were returned to cage for observation. Models were successfully established when the rats turned to the right at the time of crawling (rear‐end collision phenomenon) or even fell to the right and the right forelimb appeared adduction and flexion while the tail was lifted. In the sham group, rats were only treated with skin incision and vascular dissection without suture treatment, and the remaining steps were the same as the model group. The temperature of rats was always monitored and kept at 37℃ during the operation. After 24 hours of ischaemia, neurological function was scored with Longa assessment method for reference^19^:0 point, there was no neurological deficit; 1 point, unable to fully extend the forepaw on the paralysed side; 2 points, turning to the paralysed side when walking; 3 points, tilting to the paralysed side when walking; and 4 points, unable to walk spontaneously. Models were successfully established when neurological function scores range from 1 to 3 points. Rats were excluded if the insertion depth of the suture was less than 17 mm; if consciousness was lost (4 points) or rats died before the time of observation; and if there was subarachnoid haemorrhage or intracranial arterial thrombosis when the rat brain tissues were isolated. There was no sign of neurological injury in the sham group. According to the criteria of success and exclusion, rats failed to modelling were treated as missing values and were removed from each group. The spare rats were supplemented in the follow‐up experiments. The success rate of rat modelling was about 68.6% (120/175). Among the excluded rats, there were 4 cases whose neurological score did not meet the requirements, 3 cases died of anaesthesia accident, 2 cases with more bleeding during operation, 9 cases with difficulty in inserting the suture embolism, 4 cases died after operation, 3 cases with subarachnoid haemorrhage and 3 cases with basicranial arterial thrombosis when the brain tissues were isolated.

### 2.3 Animal grouping and treatment

Animal treatment was in line with a previous publication.^20^ Rats were divided into 8 groups (n = 15): MCAO + miR‐130a mimics group: injected with PEGliposomes‐embedded miR‐130a mimics (2.4 mg/kg, iv), given within 5 minutes after the MCAO; MCAO + mimics negative control (NC) group: injected with PEGliposomes‐embedded miR‐130a mimics NC (2.4 mg/kg, iv), given within 5 minutes after the MCAO; MCAO + miR‐130a inhibitors group: injected with PEGliposomes‐embedded miR‐130a inhibitors (2.4 mg/kg, iv), given within 5 minutes after the MCAO, given within 5 minutes after the MCAO; MCAO + inhibitors NC group: injected with PEGliposomes‐embedded miR‐130a inhibitors NC (2.4 mg/kg, iv), given within 5 minutes after the MCAO; MCAO + miR‐130a inhibitors + XIAP small interfering RNA (siRNA) group: injected with PEGliposomes‐embedded miR‐130a inhibitor (2.4 mg/kg, iv) and XIAP interference plasmids (2.4 mg/kg, iv), given within 5 minutes after the MCAO; MCAO + miR‐130a inhibitors + siRNA NC group: injected with PEGliposomes‐embedded miR‐130a inhibitors (2.4 mg/kg, iv) and NC plasmids of XIAP (2.4 mg/kg, iv), given within 5 minutes after the MCAO. MiR‐130 mimics, inhibitors, XIAP siRNA or the NCs were wrapped using PEG‐Liposome In Vivo Transfection Reagent (Altogen Biosystems, USA) and administered through the tail vein immediately after MCAO. MiR‐130 mimics, inhibitors, XIAP siRNA or the NCs were purchased from GenePharma Co., Ltd. (Shanghai, China). The validated XIAP siRNA has been confirmed to reduce XIAP expression through the functional test, and the recommended siRNA NC has been found to not target any gene product through the experimental test. Meanwhile, preliminary tests were carried out within the manufacturer to ensure the minimum target‐missing effect and siRNA delivery efficiency. XIAP siRNA knockout procedure was performed based on manufacturer's instructions. XIAP siRNA sequence was not provided due to commercial factors. Sham‐operated rats and MCAO model rats were used as controls. Rats were scored for neurological function 24 hours after ischaemia.

### 2.4 Morris water maze test

After 7 days of treatment, the spatial learning and memory ability of rats in each group were tested by Morris water maze test. The Morris water maze was 120 cm in diameter, and the circular escape platform which was 23 cm high and 10 cm in diameter was placed in the centre of the third quadrant and 2 cm below the water surface. The water temperature was kept at (24 ± 2°C) and room temperature was controlled at 24°C, and the roof and the wall surface were smooth and tidy with symmetrical lighting equipment around. The rats were placed in the water facing the pool wall from the four quadrants of the pool for the 5‐day positioning navigation test, and various parameters were recorded by the computer. If the rat did not find the platform within 2 minutes, it was then led to the platform for a 20‐seconds stay, at which time the latency was recorded as 2 minutes. On the sixth day, the platform was removed after the fifth training for the space search test. The rats were put into the water facing the pool wall from one randomly selected place of the water, and various parameters were recorded by the computer.

### 2.5 Brain tissue water content detection and haematoxylin‐eosin (HE) staining

Rats were killed by deep anaesthesia with 10% chloral hydrate (0.3 mL/100 g) and brain tissues of 5 rats in each group were taken out and immediately measured for wet weight with an electronic balance. Then the brain tissues were baked at 100°C for 48 hours and weighed for dry weight, and the water content of the brain tissue was determined by (wet weight‐dry weight)/wet weight. After another 5 rats were anaesthetized, the rats were quickly decapitated and brain tissues were taken out with the cerebellum and lower brain stem removed. Some remains were stored in liquid nitrogen, and other brain tissues 1‐4 mm behind the chiasm were obtained by coronary clip and placed in 4% paraformaldehyde to be fixed for 24 hours. Subsequently, the tissue blocks were routinely dehydrated, permeabilized, immersed in wax, embedded into wax blocks, and sliced into 5‐μm sections by coronary clip. The brain sections across the central region of the infarct were selected for staining while the brain sections at the corresponding region were selected for staining in the sham group. The sections were stained with haematoxylin for 5 minutes and differentiated with 75% hydrochloric acid‐ethanol for 30 seconds after washed by distilled water, followed by acidified eosin‐ethanol staining for 1 minute after another washing of distilled water. Finally, the sections were dehydrated, permeabilized, and sealed with neutral resin for the observation of the pathological morphology of ischaemic cerebral cortex under a light microscope.

### 2.6 Triphenyltetrazolium chloride (TTC) staining

The cerebral infarct volume was measured according to the reforming method referring to the paper written by Yin W et al^21^: tissues in each group were sectioned, stained with phosphate‐buffered saline (PBS) containing 2% TTC (Sigma, Saint Louis, MO, USA) in a 37°C incubator for 30 minutes, fixed with 4% paraformaldehyde and photographed. The infarct areas appeared white while the normal ones were red. The cerebral infarction ratio was measured by Image‐Pro Plus software (ie the percentage of the area of the infarct area to that of the homolateral cerebral hemisphere).

### 2.7 Terminal deoxyribonucleotidyl transferase‐mediated deoxyuridine triphosphate‐digoxigenin nick end labelling (TUNEL) staining

The paraffin specimens were cut into 5‐μm sections, fixed with 4% paraformaldehyde and performed with TUNEL staining in line with TUNEL kit instructions (Roche, Mannheim, Germany). The neurons in ischaemic cerebral cortex were counted by an experimental cell counter. Five random fields were selected from each group, and the apoptotic index of cells in brain tissues was calculated as (number of apoptotic cells/number of total cells) × 100%.

### 2.8 BrdU/DCX and BrdU/NeuN immunofluorescent staining

Sliced tissue blocks were heated at 60°C for 2 hours, cooled at room temperature, dewaxed and hydrated. Endogenous peroxidase was blocked with 3% H_2_O_2_ for 10 minutes. Then the sections were immersed in antigen retrieval solution ethylenediaminetetraacetic acid (EDTA)‐sodium citrate, heated with microwave for 6 minutes, naturally cooled for antigen retrieval and blocked with 10% goat serum at 37℃ for 1 hour. After the removal of excessive liquid, the sections were supplemented with primary antibody mixture (Brdu + DCX‐labelled primary antibody: mouse anti‐rat Brdu (1:50) and rabbit anti‐rat DCX (1:200), or that of Brdu + NeuN‐labelled primary antibody: mouse anti‐rat Brdu (1:50) and mouse anti‐rat NeuN (1:100)) overnight at 4°C. After 3 washes with PBS, secondary antibody mixture (Brdu + DCX‐labelled secondary antibody: fluorescein isothiocyanate (FITC)‐labelled goat anti‐mouse (1:100) and tetramethyl rhodamine iso‐thiocyanate (TRITC)‐labelled goat anti‐rabbit (1:100), or that of Brdu + NeuN‐labelled secondary antibody: FITC‐labelled goat anti‐mouse (1:100) and TRITC‐labelled goat anti‐mouse (1:100)) were added to the sections for 1‐h incubation without light exposure. Subsequently, the sections were sealed with anti‐fluorescence quencher after 3 washes by PBS again and observed under a fluorescence microscope. Six different fields were taken for each section to calculate the average number of double‐positive cells. Neural stem cells (NSCs) migrating during nerve regeneration were represented by DCX/BrdU double‐positive cells, and neurons differentiated from NSCs by NeuN/BrdU double‐positive cells.

### 2.9 CD31 and α‐smooth muscle actin (α‐SMA) immunofluorescent double staining

Sliced tissue blocks were heated at 60℃ for 2 hours, cooled, dewaxed, and hydrated. The cell membranes were ruptured with 0.25% phosphate‐buffered saline/Tween 20 (PBST) for 20 minutes. Then the sections were blocked with 2% bovine serum albumin (BSA) for 1 hour and mixed with 2% BSA‐diluted primary antibody, rabbit anti‐rat CD31 (1:200) overnight at 4℃. After rinsed with PBS 3 times, the sections were incubated with FITC‐labelled secondary antibody goat anti‐rabbit (1:100) for 2 hours without light exposure and then with 2% BSA‐diluted primary antibody, rabbit anti‐rat α‐SMA (1:200) overnight at 4°C after rinsed again. Subsequently, TRITC‐labelled secondary antibody goat anti‐rabbit (1:100) was added to the sections after 3 rinses with PBS for 2‐hour incubation at room temperature without light exposure. Finally, the sections were sealed with anti‐fluorescence quencher and observed under a fluorescence microscope. Six different fields were taken for each section to calculate the average number of double‐positive cells. The microvessel density was evaluated by new vessels expressed by CD31/α‐SMA double‐positive cells.

### 2.10 Neuron isolation, culture and identification

Culture of rat neurons: 24‐hour newborn SD rats were soaked in alcohol and the brain tissue was isolated and soaked in precooled D‐PBS balance salt solution. Meninges and blood vessels were carefully separated under a microscope. Cut into mince, tissues were detached with 0.125% trypsin at 37°C for 10 minutes, added with Dulbecco's modified Eagle medium (DMEM)/F12 containing 10% foetal bovine serum (FBS) of equal volume to stop digestion, and centrifuged at 1500 r/min for 5 minutes. The supernatant was discarded and samples were added with 3 mL DMEM/F12 containing 10% FBS again, centrifuged at 1500 r/min for 5 minutes with the supernatant removed, and added with 4 mL of neuron culture medium (98% neurobasal‐A medium + 2% B27 medium additive + 0.5 mmol/L glutamic acid + 1% penicillin‐streptomycin). Filtered with a 400‐mesh cell sieve, the cells were counted and seeded onto plates coated with polylysine. After 4‐hour culture, the whole solution was replaced by neuron culture solution and from that on, the half medium was changed every 3 d.

Identification of rat neurons: 10‐d neurons were fixed with 4% paraformaldehyde for 15 minutes, treated with 0.3% Triton X‐100 for 10 minutes, blocked with 3% BSA for 30 minutes with medium discarded, and incubated with microtubule‐associated protein 2 (MAP‐2) antibody (1:100) overnight at 4°C. Next, the neurons were incubated with secondary antibody immunoglobulin G Cy3 (1:100) without light exposure for 2 hours, sealed and observed and photographed under an inverted fluorescent microscope.

### 2.11 Cell grouping and treatment

Cell grouping: the control group (neurons without transfection), the oxygen‐glucose deprivation (OGD) group (neurons were treated with OGD), the OGD + miR‐130a mimics group (neurons were transfected with miR‐130 mimics before exposure to OGD for 24 hours), OGD + mimics NC group (neurons were transfected with miR‐130 mimics NC before exposure to OGD for 24 hours), the OGD + miR‐130a inhibitors group (neurons were transfected with miR‐130 inhibitors before exposure to OGD for 24 hours), the OGD + inhibitors NC group (neurons were transfected with miR‐130 inhibitors NC before exposure to OGD for 24 hours), the OGD + miR‐130a inhibitors + XIAP siRNA group (neurons were transfected with miR‐130 inhibitors and XIAP siRNA before exposure to OGD for 24 hours) and the OGD + miR‐130a inhibitors + siRNA NC group (neurons were transfected with miR‐130 inhibitors and XIAP siRNA NC before exposure to OGD for 24 hours).

Oligonucleotides or siRNA was purchased from GenePharma. P3 cells were trypsinized and seeded onto 6‐well plates at 3 × 10^6^ cells/well. The cells were replaced by serum‐free medium for 1‐h incubation when cell confluence reached 60%, and then were respectively transfected by Lipofectamine 2000 reagent (Invitrogen).

OGD modelling: the hypoxic medium was treated in a hypoxic incubator with 1% O_2_, 5% CO_2_, and 94% N_2_ (Thermo Fisher Scientific) for 30 minutes. Cells were cultured in the medium as previously described.^22^ Cells in the control group were placed in fresh extracellular matrix in an incubator with 21% CO_2_ and 79% O_2_ (normoxic condition).

### 2.12 3‐(4,5‐dimethyl‐2‐thiazolyl)‐2,5‐diphenyl‐2‐H‐tetrazolium bromide (MTT) assay

Cells were incubated in MTT solution for 4 hours, treated with dimethyl sulfoxide (both from Sigma) and shaken for 10 minutes to dissolve the crystal. The optical density (OD) value at 490 nm was analysed using a full‐automatic microplate reader (Beckman Coulter Inc, MN, USA). The cell viability = OD value of the experimental group/OD value of the control group × 100%.

### 2.13 Flow cytometry

Trypsinized cells were resuspended with 100 μL 1 × binding buffer to prepare single cell suspension (1 × 10^6^ cells/mL). The suspension was treated with 2 μL Annexin‐V‐FITC (20 μg/mL) on ice with light avoidance for 15 minutes, transferred into flow tubes and added with 300 μL PBS. Each sample was added with 1 μL PI (50 μg/mL) and detected by a flow cytometer (Beckman Coulter Inc) in 30 minutes.

### 2.14 Reverse transcription quantitative polymerase chain reaction (RT‐qPCR)

The total RNA was extracted from tissues and cells by TRIzol (Invitrogen, California, USA), stored at −80°C, then reversely transcribed into cDNA by PrimeSeript@RT reagent Kit (TaKaRa, Dalian, China) and stored at −20°C for further experiment. U6 and glyceraldehyde phosphate dehydrogenase (GAPDH) were taken as the internal reference of miR‐130a and other genes respectively. RT‐qPCR assay was performed by ABI7500 quantitative PCR instrument (Applied Biosystems, CA, USA) and relative quantification was performed using the comparative CT (2‐DeltaDeltaCT) method. The primers used are displayed in Table 1. Each sample was measured 3 times repeatedly.

**Table 1:** RT‐qPCR primer sequence

Gene	Primer sequence
miR‐130a	Forward: 5’‐CTTGAAGGTAATGGAACCCGG‐3’
	Reverse: 5’‐GTGATACCCGTGGTAGTACCTC‐3’
U6	Forward: 5’‐AGGACTATCGTCCGATCAAC‐3’
	Reverse: 5’‐GCCCTCACACCTGCACTGTGTC‐3’
XIAP	Forward: 5’‐CCCTTGGGAACAGCATGCTA‐3’
	Reverse: 5’‐AATCCAGCACCACAGTAGGC‐3’
BDNF	Forward: 5’‐CTGGAGAAACTCCCGGTATC‐3’
	Reverse: 5’‐GGTAGTTCGGCATTGCGAGT‐3’
NGF	Forward: 5’‐TCGAGGCACACAAAGAAGA‐3’
	Reverse: 5’‐TCACCGCATGGGTTAAAG‐3’
VEGF	Forward: 5’‐TGCACCCACGACAGAAGGGGA‐3’
	Reverse: 5’‐TCACCGCCTTGGCTTGTCACAT‐3’
HGF	Forward: 5’‐TCTTGACCCTGACACCCC‐3’
	Reverse: 5’‐GTGATTCAGCCCCATCCGG‐3’
GAPDH	Forward: 5’‐ACCACAGTCCATGCCATCAC‐3’
	Reverse: 5’‐TCCACCACCCTGTTGCTGTA‐3’

Abbreviations: BDNF, brain‐derived neurotrophic factor; GAPDH, glyceraldehyde‐3‐phosphate dehydrogenase; HGF, hepatocyte growth factor; miR‐130a, microRNA‐130a; NGF, nerve growth factor; VEGF, vascular endothelial growth factor; XIAP, X‐linked inhibitor of apoptosis protein.

### 2.15 Western blot analysis

Total proteins from tissues and cells were extracted, and the concentrations were determined using a bicinchoninic acid kit (Qiagen GmbH, Hilden, Germany) according to the manufacturer's protocol. Total protein of 60 μg was electrophoresed with gel at 70 V for 120 minutes, then transferred to polyvinylidene fluoride membrane, blocked for 1.5 hours with 5% BSA and incubated with primary antibodies (XIAP, 1:500, ab2541; Caspase‐3, 1:1000, ab13847; B‐cell lymphoma‐associated x (Bax), 1:1000, ab32503; B‐cell lymphoma‐2 (Bcl‐2), 1:1000, ab196495; brain‐derived neurotrophic factor (BDNF), 1:500, ab226843; nerve growth factor (NGF), 1:500, ab6199; vascular endothelial growth factor (VEGF), 1:600, sc‐7269; hepatocyte growth factor (HGF), 1:500, ab83760; GAPDH, 1:1000, ab181602) for 2 hours and then overnight at 4℃. All the antibodies were purchased from Abcam (Cambridge, MA, USA) and Santa Cruz Biotechnology (Santa Cruz, California, USA). The membrane was washed 3 times with tris‐buffered saline with Tween 20 (TBST) and incubated with horseradish peroxidase‐labelled secondary antibody for 1.5 hours at room temperature. The bands were stained by chemiluminescence reagent, and the grey value of protein expression was analysed by Image software. GAPDH was used as an internal reference, and the relative content of the target protein was expressed by the ratio of the density of the target protein to that of GAPDH.

### 2.16 Dual‐luciferase reporter gene assay

MiR‐130a's target gene was evaluated using the TargetScan database to verify if miR‐130a was able to target and inhibit XIAP. The 3’‐UTR of XIAP containing the putative miR‐130a binding sequence was amplified by PCR and cloned downstream of the firefly luciferase gene in the pMIR‐Report vector (Promega Corporation, Madison, WI, USA) to construct pMIR‐XIAP‐3’‐UTR‐ Wt vector (XIAP‐Wt). Mutations in the miR‐130a seed regions of the XIAP 3’UTR were generated using the QuikChange Multisite‐directed mutagenesis kit (Promega Corporation). The mutated XIAP 3’‐UTR fragment was cloned into the pMIR‐Report vector to develop the pMIR‐XIAP −3’‐UTR‐Mut vector(XIAP‐Mut). MiR‐130a mimics with NC sequence and sequenced luciferase reporter vector (XIAP‐Wt and XIAP‐Mut) were co‐transfected into HEK293T cells respectively and luciferase activity was detected according to the method provided by Promega company (Madison, Wisconsin, USA).

### 2.17 Statistical analysis

The data were analysed by statistical software SPSS 21.0 (IBM Corp., Armonk, NY, USA) and found normally distributed by Kolmogorov‐Smirnov test. The results were expressed as mean ± standard deviation. Comparisons between two groups were analysed by t test while comparisons among groups by one‐way analysis of variance (ANOVA). The pairwise comparisons were performed with Tukey's post hoc test. *P* was tested bilaterally, and the difference was statistically significant at *P* < 0.05.

## 3 RESULTS

### 3.1 MiR‐130a is up‐regulated and XIAP is down‐regulated in MCAO rats; miR‐130a inhibits XIAP

**Figure 1:**
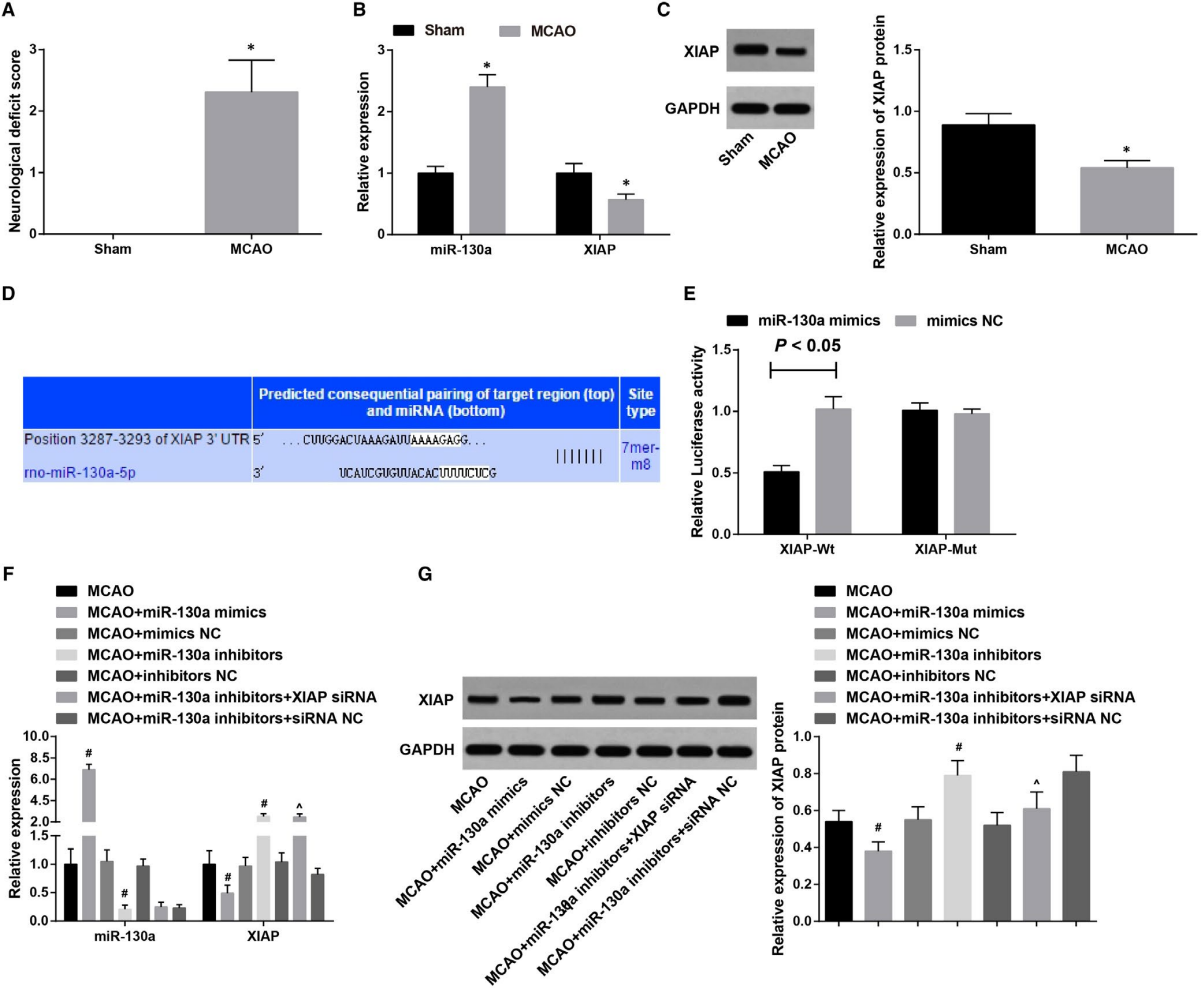
MiR‐130a is up‐regulated and XIAP is down‐regulated in MCAO rats; miR‐130a inhibits XIAP expression. A, Neurological impairment scores of MCAO rats; B, Detection of miR‐130a and XIAP expression in brain tissues of rats in the sham and MCAO groups by RT‐qPCR; C, Detection of XIAP protein expression in brain tissues of rats in the sham and MCAO groups by Western blot analysis; D, Binding site between miR‐130a and XIAP was predicated by bioinformatics tool; E, Verification of the binding site between XIAP 3'‐UTR and miR‐130a by dual‐luciferase reporter gene assay; F, Detection of miR‐130a and XIAP expression in brain tissues of treated MCAO rats by RT‐qPCR; G, Detection of XIAP protein expression in brain tissues of treated MCAO rats by Western blot analysis. *P < 0.05 vs the sham group; ^#^ *P* < 0.05 vs the MCAO group; ^&^ *P* < 0.05 vs the MCAO + miR‐130a inhibitors group. The results were expressed as mean ± standard deviation, and comparisons between two groups were analysed by t test while comparisons among groups by one‐way ANOVA, followed by Tukey's post hoc test

Rat neurological impairment was scored 24 hours after ischaemia to clarify whether the modes were successfully constructed (Figure 1A). It was found that rats in the sham group exhibited no neurological impairment and the score was 0; MCAO rats had severe neurological impairment and increased scores, indicating a successful modelling. Outcomes of RT‐qPCR and Western blot analysis (Figure 1B,C) showed that MCAO rats had up‐regulated miR‐130a and down‐regulated XIAP versus those in the sham group (both *P* < 0.05). Through searching the TargetScan database, we found that XIAP was a potential target gene of miR‐130a (Figure 1D). It was further confirmed that co‐transfection of miR‐130a mimics and XIAP‐Wt suppressed the luciferase activity (*P* < 0.05), while the co‐transfection of miR‐130a mimics and XIAP‐Mut did not affect the luciferase activity (*P* > 0.05). The luciferase activity did not obviously vary in the mimics NC co‐transfection groups (*P* > 0.05). These data indicate that miR‐130a targets XIAP (Figure 1).

Results of RT‐qPCR and Western blot analysis after interference (Figure 1F,G) revealed that miR‐130a and XIAP expression showed no significant difference among the MCAO, MCAO + mimics NC and MCAO + inhibitors NC groups (both *P* > 0.05). MiR‐130a mimics up‐regulated miR‐130a and down‐regulated XIAP in MCAO rats, while miR‐130a inhibitors had totally opposite effects on miR‐130a and XIAP expression in MCAO rats (both *P* < 0.05). Versus the MCAO + miR‐130a inhibitors group, miR‐130a and XIAP expression had no broad difference in the MCAO + miR‐130a inhibitors + siRNA NC group (both *P* > 0.05); miR‐130a did not markedly change (*P* > 0.05) and XIAP was down‐regulated (*P* < 0.05) in the MCAO + miR‐130a inhibitors + XIAP siRNA group.

### 3.2 Inhibition of miR‐130a improves neurological deficit in MCAO rats

**Figure 2:**
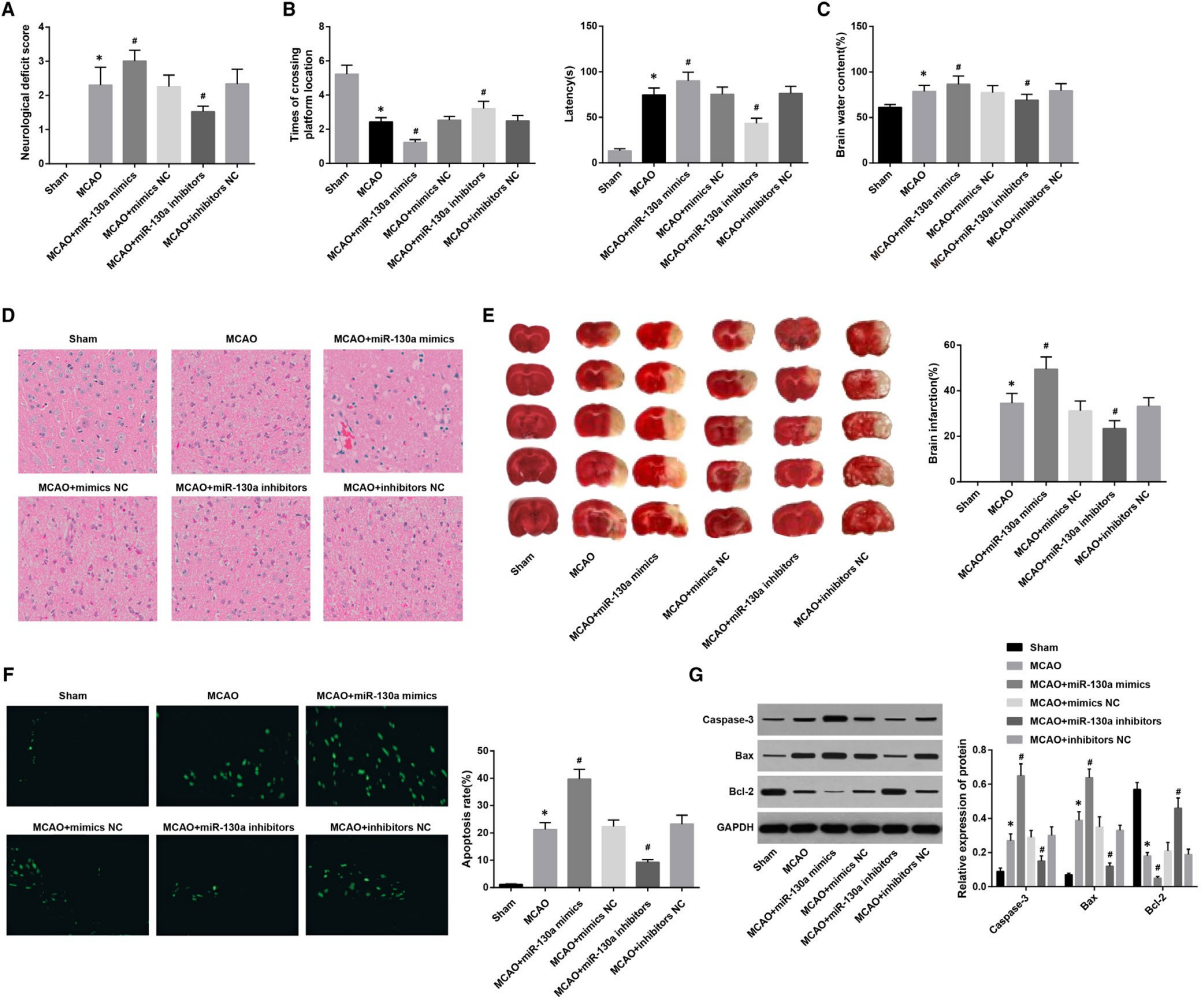
Suppression of miR‐130a improves neurological deficit in rats with MCAO. A: Neurological deficit scores of rats in each group; B, Comparisons of spatial learning and memory function (latency and times of crossing the platforms) of rats in each group; C, Water content of brain tissues of rats in each group; D, HE staining of the brain tissues of rats in each group (× 400); E, Comparisons of TTC staining and MCAO ratio in the brain tissues in each group; F, Comparisons of TUNEL staining and apoptotic index in the brain tissues in each group (× 400); G, Expression of Caspase‐3, Bax and Bcl‐2 detected by Western blot analysis. *P < 0.05 vs the sham group; ^#^ *P* < 0.05 vs the MCAO group. The results were expressed as mean ± standard deviation and comparisons among groups by one‐way ANOVA, followed by Tukey's post hoc test

The neurological function scores of the rats in each group were observed 24 hours after ischaemia. The results revealed (Figure 2A) that the MCAO group had higher scores than the sham group. In contrast to the MCAO group, scores of the MCAO + miR‐130a mimics group rose dramatically, and scores of the MCAO + miR‐130a inhibitors group noticeably reduced (all *P* < 0.05), while scores of the MCAO + mimics NC group and the MCAO + inhibitors NC group were not obviously different (*P* > 0.05).

Seven days after grouping, the spatial learning and memory function of the rats were detected. The results indicated that (Figure 2B) there were prolonged latency and significantly reduced times of crossing the platform in the MCAO group in contrast to the sham group (all *P* < 0.05). Versus the MCAO group, prolonged latency and decreased times of crossing the platform occurred in the MCAO + miR‐130a mimics group while shortened latency and increased times of crossing the platform in the MCAO + miR‐130a inhibitors group (all *P* < 0.05). The latency and times of crossing the platform in the MCAO + mimics NC group and MCAO + inhibitors NC group exhibited no difference (both *P* > 0.05). It is suggested that inhibited miR‐130a can improve the learning and memory ability of rats with MCAO.

Brain tissue water content test revealed that (Figure 2C) water content in brain tissues in the sham group was lower than the MCAO group (*P* < 0.05). The water content in brain tissues was higher in the MCAO + miR‐130a mimics group and lower in the MCAO + miR‐130a inhibitors group versus that in the MCAO group (all *P* < 0.05). The brain tissue water content showed no apparent difference between the MCAO + mimics NC and MCAO + inhibitors NC groups (*P* > 0.05).

HE staining indicated that (Figure 2D) the neurons in the cerebral cortex of the sham group densely arranged and had integrate morphology, large and round nuclei with clear outline, and obvious nucleolus. In the MCAO, MCAO + mimics NC and MCAO + inhibitors NC groups, neurons in the ischaemic cortex had atrophic morphology, accompanied by nuclear pyknosis, fragmentation and dissolution. In comparison with the MCAO group, there were more pathological changes in ischaemic cerebral cortex tissues in the MCAO + miR‐130a mimics group: large area liquefying necrotic focus, inflammatory infiltration and neuronal atrophy and dissolution. Neurons in the MCAO + miR‐130a inhibitors group showed mild oedema, complete outline and obvious nucleolus, and a small number of neurons exhibited atrophic morphology and karyopyknosis.

TTC staining showed (Figure 2E) that in the sham group, the tissue in the brain hemisphere on the ischaemic side was red, suggesting no infarction, while there were white areas showing the appearance of infarction in the MCAO group. Versus the MCAO group, the brain infarction rate markedly rose in the MCAO + miR‐130a mimics group and dramatically declined in the MCAO + miR‐130a inhibitors group (*P* < 0.05). The brain infarction rate was almost the same as that in the MCAO + mimics NC group and MCAO + inhibitors NC group (*P* > 0.05).

TUNEL staining showed (Figure 2F) that there was little TUNEL‐positive cells expression in the sham group; however, the apoptotic index in the MCAO group was higher than the sham group (*P* < 0.05). Versus the MCAO group, the MCAO + miR‐130a mimics had higher apoptotic index, while the MCAO + miR‐130a inhibitors group had lower apoptotic index (both *P* < 0.05). The apoptotic index showed no considerable difference between the MCAO + mimics NC and MCAO + inhibitors NC groups (*P* > 0.05).

Western blot analysis revealed (Figure 2G) that apoptosis‐related protein (Caspase‐3 and Bax) expression dramatically increased and Bcl‐2 expression markedly diminished in the MCAO group relative to the sham group (all *P* < 0.05). Versus the MCAO group, Caspase‐3 and Bax expression rose greatly and Bcl‐2 expression greatly declined in the MCAO + miR‐130a mimics group, while Caspase‐3 and Bax expression diminished and Bcl‐2 expression rose in the MCAO + miR‐130a inhibitors group (all *P* < 0.05), but Caspase‐3, Bax and Bcl‐2 expression in the MCAO + mimics NC group and MCAO + inhibitors NC group showed no significance (all *P* > 0.05). It is suggested that suppression of miR‐130a can improve nerve damage in MCAO rats.

### 3.3 Suppressed miR‐130a promotes the proliferation and differentiation of NSCs in the brain tissues in MCAO rats

**Figure 3:**
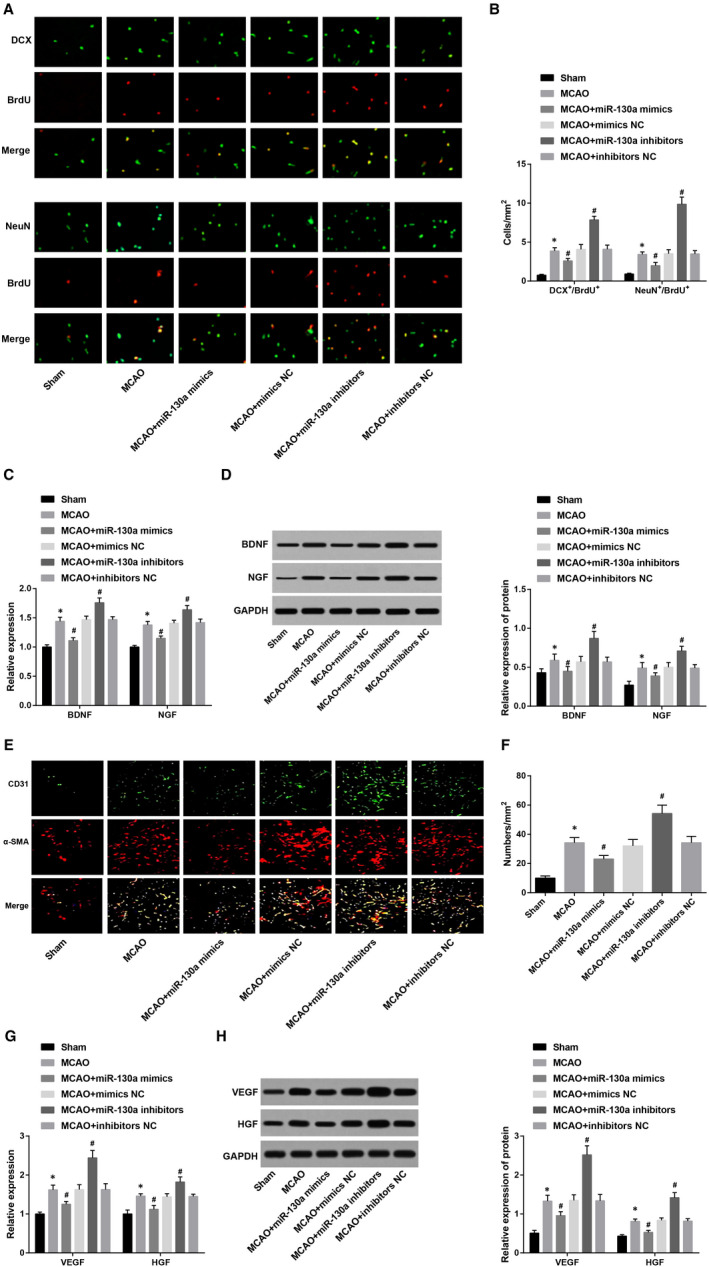
Inhibition of miR‐130a promotes the proliferation and differentiation of NSCs in the brain tissues in rats with MCAO. A, Proliferation and differentiation of endogenous NSCs in the brain tissues by double‐labelling immunofluorescence assay; B. Quantification results in Panel A; C & D, Detection of neurotrophic factor (BDNF and NGF) expression by RT‐qPCR and Western blot analysis; E, Evaluation of the microvessel density in the brain infarction area by immunofluorescence staining; F, Statistical results of Panel E; G & H, Detection of angiogenesis‐related factor (VEGF and HGF) expression by RT‐qPCR and Western blot analysis. *P < 0.05 vs the sham group; ^#^ *P* < 0.05 vs the MCAO group. The results were expressed as mean ± standard deviation and comparisons among groups by one‐way ANOVA, followed by Tukey's post hoc test

Double‐labelling immunofluorescence assay (Figure 3A,B) indicated that the numbers of DCX/BrdU double‐positive cells and NeuN/BrdU double‐positive cells were reduced in ischaemic cerebral cortex tissues of the sham group, which was less than those in the MCAO group. However, compared to the MCAO group, the numbers of DCX/BrdU double‐positive cells and NeuN/BrdU double‐positive cells were decreased in the MCAO + miR‐130a mimics group and further increased in the MCAO + miR‐130a inhibitors group (all *P* < 0.05). The numbers of DCX/BrdU double‐positive cells and NeuN/BrdU double‐positive cells exhibited no remarkable difference among the MCAO, MCAO + mimics NC and MCAO + inhibitors NC groups (all *P* > 0.05).

Neurotrophin (BDNF and NGF) expression was detected (Figure 3C,D). It was shown that the MCAO rats had higher BDNF and NGF expression than those in the sham group, and this increase in BDNF and NGF expression in MCAO rats was suppressed by miR‐130a mimics, while was promoted by miR‐130a inhibitors (all *P* < 0.05). There was no obvious difference in BDNF and NGF expression among the MCAO, MCAO + mimics NC and MCAO + inhibitors NC groups (both *P* > 0.05).

It has been validated that angiogenesis plays a vital role in neurological functional restoration after cerebral infarction.^23^ In the present study, we selected CD31 as the endothelial cell marker and α‐SMA as the pericyte marker, and the cerebral ischaemic cortex penumbra region was performed with CD31 and α‐SMA immunofluorescent double staining to measure the microvessel density. We discovered that (Figure 3E,F) there were fewer new vessels occurring in the brain tissues of the sham group, but a large number around the lesion in the MCAO group (CD31/α‐SMA double positive). In contrast to the MCAO group, the number of new vessels was diminished in the MCAO + miR‐130a mimics group, and was increased in the MCAO + miR‐130a inhibitors group (both *P* < 0.05). The number of new vessels showed no evident difference among the MCAO, MCAO + mimics NC and MCAO + inhibitors NC groups (*P* > 0.05).

VEGF and HGF expression was detected (Figure 3G,H). Compared with the sham group, VEGF and HGF expression was increased in the MCAO group (both *P* < 0.05). VEGF and HGF expression was diminished in the MCAO + miR‐130a mimics group and was increased in the MCAO + miR‐130a inhibitor group versus the MCAO group (all *P* < 0.05). Apparent difference could not be found in VEGF and HGF expression among the MCAO, MCAO + mimics NC and MCAO + inhibitors NC groups (both *P* > 0.05). These results illustrate that the inhibited miR‐130a promotes the proliferation and differentiation of NSCs in the brain tissue in MCAO rats.

### 3.4 Inhibited XIAP reverses the effect of inhibited miR‐130a on neurological function and angiogenesis in MCAO rats

**Figure 4:**
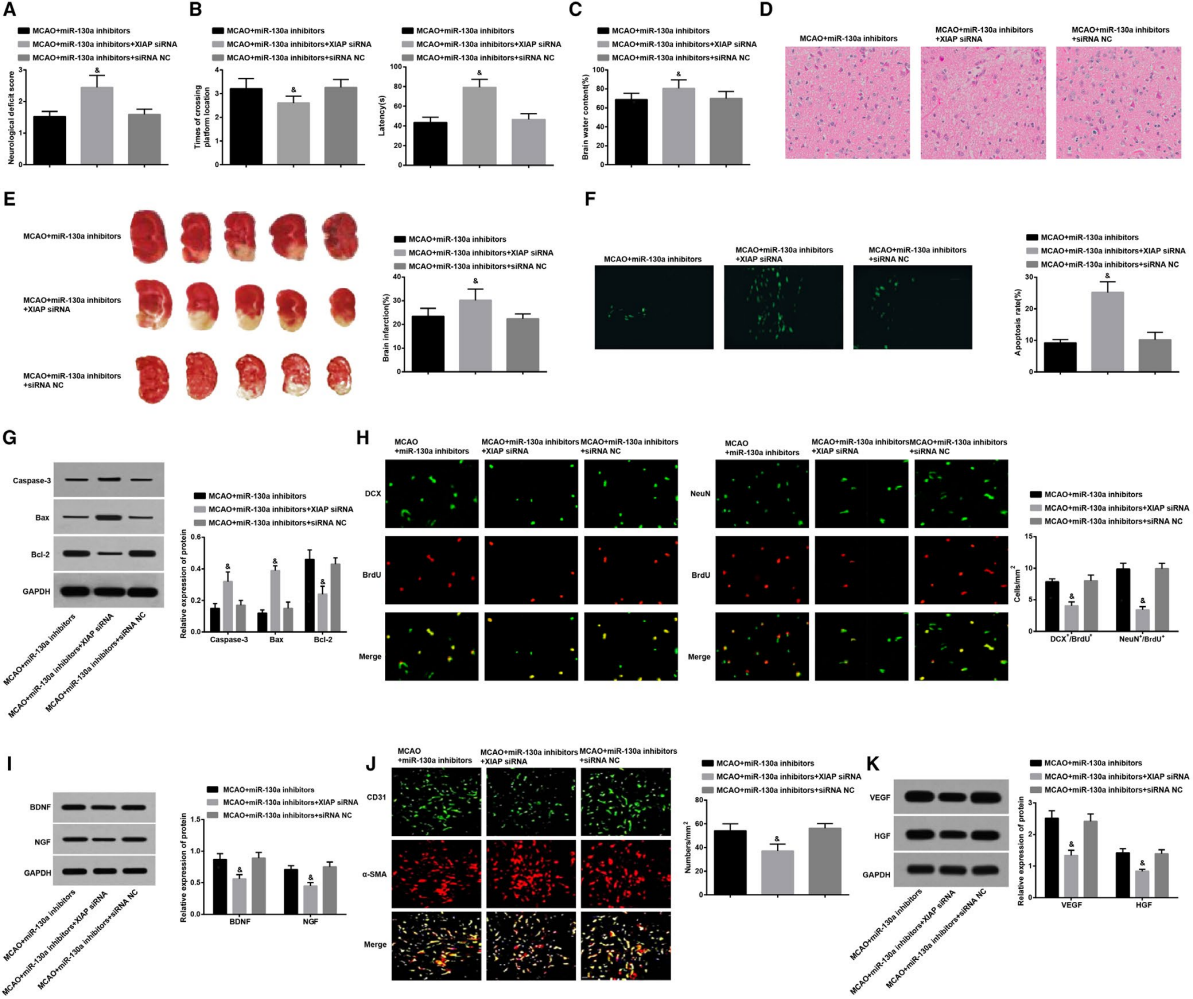
Inhibited XIAP reverses the effect of inhibited miR‐130a on neurological function and angiogenesis in rats with MCAO. A, Neurological function scores in each group; B, Spatial learning and memory function (latency and times of crossing the platforms) of rats in each group; C, Water content of brain tissue in each group; D, HE staining of brain tissues in each group (× 400); E, Comparisons of TTC staining and infarction ratio in the brain tissues in each group; F, Comparisons of TUNEL staining and the apoptotic index in the brain tissues in each group of rats (× 400); G, Expression of Caspase‐3, Bax and Bcl‐2 detected by Western blot analysis; H, Detection of proliferation and differentiation of endogenous NSCs in the brain tissues by double‐labelling immunofluorescence assay as well as its statistics analysis; I, Expression of neurotrophic factors (BDNF and NGF) in the brain tissues in each group; J, Evaluation of microvessel density in the brain infarction area by immunofluorescence staining; K, Expression of VEGF and HGF in the brain tissues in each group; ^#^ *P* < 0.05 vs the MCAO + miR‐130a inhibitors group. The results were expressed as mean ± standard deviation and comparisons among groups by one‐way ANOVA, followed by Tukey's post hoc test

In contrast to the MCAO + miR‐130a inhibitors group, there was reduced XIAP expression in the MCAO + miR‐130a inhibitors + XIAP siRNA group, along with increased neurological function score, latency, water content of brain tissues, aggravated pathological changes, increased number of necrotic cells, apoptotic index, incremental cerebral infarction rate and Caspase‐3 and Bax expression, and decreased times of crossing platforms, Bcl‐2, BDNF and NGF expression, DCX/BrdU and NeuN/BrdU double‐positive cells, VEGF and HGF expression, and new blood vessels (all *P* < 0.05). Nevertheless, there was no marked change in all corresponding indices in the MCAO + miR‐130a inhibitors + siRNA NC group versus the MCAO + miR‐130a inhibitors group (all *P* > 0.05) (Figure 4A‐K). These findings reveal that inhibition of XIAP can reverse the effect of suppressed miR‐130a on neurological function and angiogenesis in rats with MCAO.

### 3.5 Suppressed miR‐130a promotes neuron viability and decelerates apoptosis in a cell model of OGD, and reduced XIAP abolished these effects

**Figure 5:**
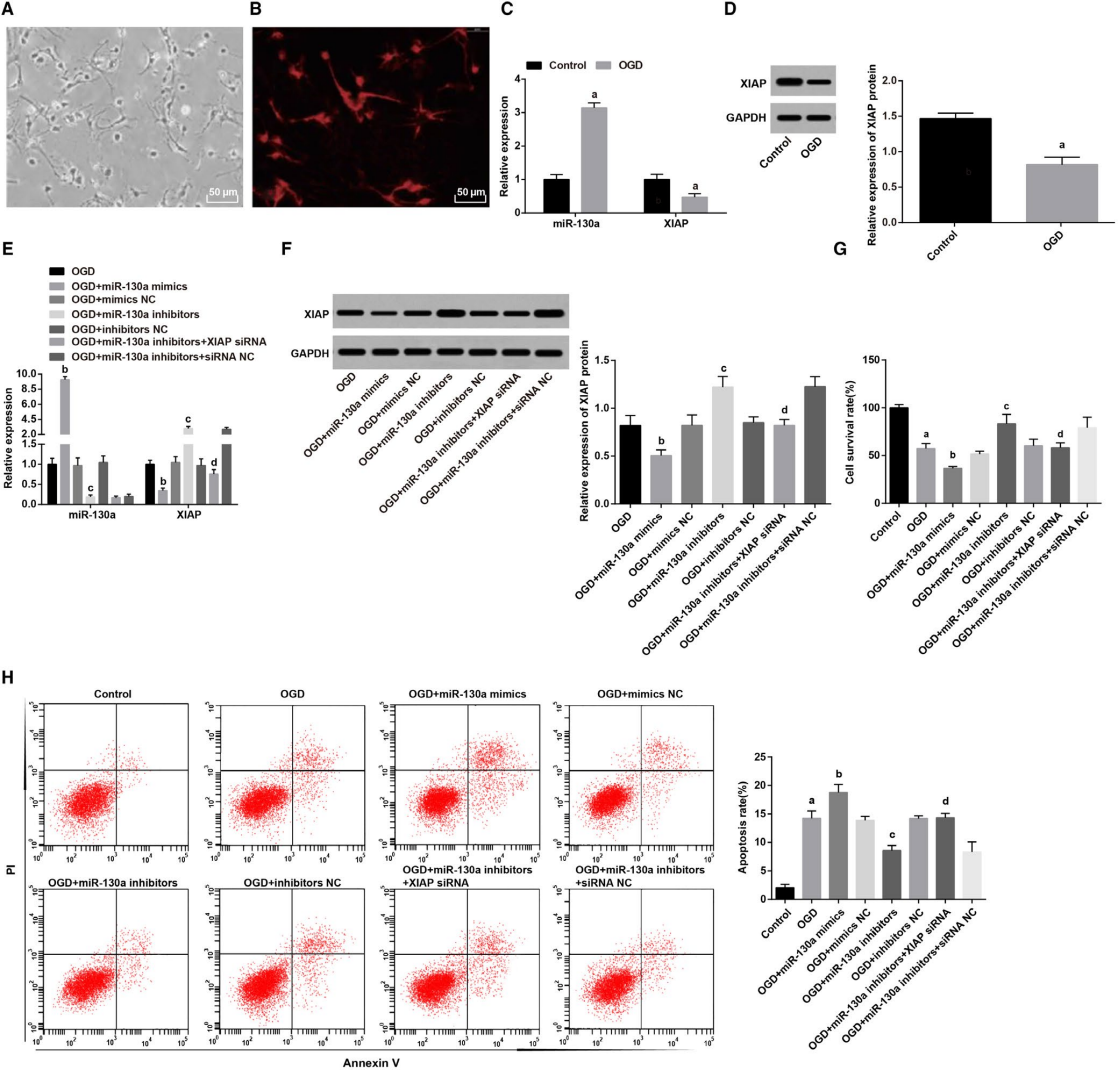
Suppressed miR‐130a promotes neuron viability and decelerates apoptosis of in a cell mode of OGD, and reduced XIAP abolished these effects. A, Primary neurons (× 200); B, Neurons were identified by immunohistochemical staining (× 200); C, miR‐130a and XIAP expression in neurons was determined using RT‐qPCR; D, XIAP protein expression in neurons was assessed by Western blot analysis; E, The neuron viability was measured by MTT assay; F, Flow cytometry was used to assess the apoptosis rate of neurons. a, *P* < 0.05 vs the control group; b, *P* < 0.05 vs the OGD group; c, *P* < 0.05 vs the OGD + miR‐130a inhibitors group. The results were expressed as mean ± standard deviation and comparisons among groups by one‐way ANOVA, followed by Tukey's post hoc test

The rat primary neurons cultured for 5‐6 d were observed. The cells were triangular, fusiform and irregular with well stereopsis and refraction. The processes were extremely developed and connected with each other to form dense network of nerve fibres. The background was clean (Figure 5A). MAP‐2 was used to identify the neurons cultured for 10 d. The MAP‐2 specific protein labelled by Cy3 expressed in the cytoplasm of neurons and was stained into orange red. It could be seen that the morphology of cultured neurons was typical and the cells were connected into a network (Figure 5B), suggesting that the cultured neurons had well purity and could be used in subsequent experiments.

MiR‐130a and XIAP expression in neurons was determined. We found that (Figure 5C,D) the neurons in the OGD group had higher miR‐130a expression and lower XIAP expression than those in the control group (both *P* < 0.05). miR‐130a and XIAP expression exhibited no marked difference among the OGD, OGD + mimics NC and OGD + inhibitors NC groups (both *P* > 0.05). miR‐130a mimics up‐regulated miR‐130a and down‐regulated XIAP, and miR‐130a inhibitors had opposite effects on miR‐130a and XIAP expression (both *P* < 0.05). Versus the OGD + miR‐130a inhibitors group, miR‐130a and XIAP expression did not evidently change in the OGD + miR‐130a inhibitors + siRNA NC group (both *P* > 0.05), miR‐130a expression presented no obvious difference (*P* > 0.05) while XIAP was down‐regulated (*P* < 0.05) in the OGD + miR‐130a inhibitors + XIAP siRNA group.

The neuron viability and apoptosis were assessed. The results (Figure 5E,F) implied that OGD‐treated neurons had reduced viability and promoted apoptosis rate versus those in the control group (both *P* < 0.05). The viability and apoptosis rate showed no remarkable difference among the OGD, OGD + mimics NC and OGD + inhibitors NC groups (both *P* > 0.05). In a cell model of OGD, miR‐130a mimic suppressed neuron viability and promoted apoptosis rate, whereas miR‐130a inhibitors exerted reverse effect on neuron viability and apoptosis rate (both *P* < 0.05). Contrasted to the OGD + miR‐130a inhibitors group, neuron viability and apoptosis rate did not change in the OGD + miR‐130a inhibitors + siRNA NC group (both *P* > 0.05), but the neuron viability was suppressed and the apoptosis rate was elevated in the OGD + miR‐130a inhibitors + XIAP siRNA group (both *P* < 0.05).

## 4 DISCUSSION

Ischaemic stroke, the most universal kind of stroke, may arise while a blood vessel in the brain is occluded.^24^ Among numerous factors that result in ischaemic stroke, gender and age are the most influential ones, followed by other factors such as alcoholism and smoking.^25^ In recent years, miRNAs have been proposed to be tightly correlated with ischaemic stroke.^26^ Importantly, miR‐130a was implicated in ischaemic stroke^10^ and cerebral ischaemia‐induced blood‐brain barrier permeability.^11^ Nevertheless, the role of miR‐130a targeting XIAP in ischaemic stroke remains largely unknown. Thus, we aim to explore the role of miR‐130a in the neurological deficit and angiogenesis in MCAO rats via targeting XIAP. Collectively, our study unravelled that miR‐130a regulated neurological deficit and angiogenesis in rats with MCAO by targeting XIAP.

One significant finding in our study is that miR‐130a was up‐regulated while XIAP was down‐regulated in MCAO rats. Concurring with our study, a previous study has suggested that miR‐130a is overexpressed in rats with transient MCAO when hypoxia occurs and in acute ischaemic stroke patients’ serum,^11^ and Wang et al have found that high serum miR‐130a levels were associated with severe perihematomal oedema and predicted adverse outcome in acute intracerebral haemorrhage.^27^ There has been a study revealing that XIAP is declined in rats suffering from cerebral ischaemia reperfusion injury,^28^ and a previous study has clarified that XIAP protein levels were decreased in both sexes after stroke.^15^ Additionally, this study showed that miR‐130a inhibited XIAP expression, and XIAP was a target gene of miR‐130a. Similarly, it is indicated that miR‐130a targets XIAP so as to regulate cisplatin chemosensitivity in the cells ovarian cancer.^16^

Brain‐derived neurotrophic factor is a neurotrophin which exerts significant functions on development and neuroplasticity of neurons and changes in BDNF expression are implicated in various psychiatric and neurological disorders.^29^ NGF is neuroprotective, namely capable of diminishing cell death in vitro usually determined within the first 48 hours’ plating.^30^ Thus, we detected expression of BDNF and NGF in brain tissues of MCAO rats to reveal the roles of miR‐130a and XIAP in biological functions of NSCs. Our results indicated that down‐regulated miR‐130a suppressed angiogenesis and improved neurological deficit and NSC proliferation and differentiation in the brain tissues of MCAO rats. The in vitro assays indicated that miR‐130a inhibition promoted neuron viability and decelerated apoptosis in a cell model of OGD. Similarly, it has been proposed by Yang et al that exosome‐derived miR‐130a activated angiogenesis in gastric cancer in vascular endothelial cells,^31^ and a recent study has revealed that lithium‐containing biomaterials stimulated bone marrow stromal cell‐derived exosomal miR‐130a secretion to promote angiogenesis.^32^ Moreover, miR‐130a has also been unravelled to increase osteogenic differentiation and attenuate adipogenic differentiation of bone marrow mesenchymal stem cells.^33^ In addition, Li et al have found that miR‐130a inhibition protected rat cardiac myocytes from hypoxia‐triggered apoptosis,^34^ and it has been uncovered that miR‐130a alleviated neuronal apoptosis and changes in expression of Bcl‐2/Bax and Caspase‐3 in cerebral infarction rats.^35^

We conducted further experiments to discover that inhibited XIAP reversed the effect of suppressed miR‐130a on MCAO rats and OGD‐treated neurons. As previously described, up‐regulated XIAP can lead to cell death in hippocampal cultures exposed to OGD, prevention of oxidative stress, cytochrome *c* release reduction, and decrease of injury in males that suffered from stroke.^15^ In line with our study, XIAP has been validated to suppress Caspase‐3 together with Caspase‐7 employing its 2nd baculovirus IAP repeat domain.^36^ It has been also verified that XIAP can increase BDNF expression via the modulation of NF‐κB.^37^

In conclusion, our study verified that miR‐130a regulated neurological deficit and angiogenesis in rats with MCAO by targeting XIAP. Our findings provide new clues for the role of miR‐130a/XIAP axis in MCAO rats and create new inspirations for the treatment of ischaemic stroke, which is of great realistic significance. However, further research is expected to better elucidate the impacts of miR‐130a on ischaemic stroke.

## CONFLICT OF INTEREST

The authors declare that they have no conflicts of interest.

## AUTHOR CONTRIBUTION

**Wenjing Deng:** Writing‐review & editing (equal). **Chenghe Fan:** Writing‐review & editing (equal). **Yanan Zhao:** Investigation (equal); Methodology (equal). **Yuewei Mao:** Investigation (equal); Methodology (equal). **Jiajia Li:** Data curation (equal); Formal analysis (equal). **Yonggan Zhang:** Data curation (equal); Formal analysis (equal). **Junfang Teng:** Conceptualization (equal).

## Data Availability

The data that support the findings of this study are available from the corresponding author upon reasonable request.

## References

[1] Goyal M , Demchuk AM , Menon BK , et al. Randomized assessment of rapid endovascular treatment of ischemic stroke. N Engl J Med. 2015;372(11):1019‐1030. 25671798 10.1056/NEJMoa1414905

[2] Papadopoulos CM , Tsai S‐Y , Cheatwood JL , et al. Dendritic plasticity in the adult rat following middle cerebral artery occlusion and Nogo‐a neutralization. Cereb Cortex. 2006;16(4):529‐536. 16033928 10.1093/cercor/bhi132

[3] Xu YL , et al. Aldehyde dehydrogenase 2 rs671G>A polymorphism and ischemic stroke risk in Chinese population: a meta‐analysis. Neuropsychiatr Dis Treat. 2019;15:1015‐1029. 31114208 10.2147/NDT.S196175 PMC6497503

[4] Cohen JE , Leker RR . Endovascular treatment for acute ischemic stroke. N Engl J Med. 2013;368(25):2432. 10.1056/NEJMc1304759 23782187

[5] Feng X , Wang Z , Fillmore R , et al. MiR‐200, a new star miRNA in human cancer. Cancer Lett. 2014;344(2):166‐173. 24262661 10.1016/j.canlet.2013.11.004 PMC3946634

[6] Hansen TB , Wiklund ED , Bramsen JB , et al. miRNA‐dependent gene silencing involving Ago2‐mediated cleavage of a circular antisense RNA. EMBO J. 2011;30(21):4414‐4422. 21964070 10.1038/emboj.2011.359 PMC3230379

[7] Ha M , Kim VN . Regulation of microRNA biogenesis. Nat Rev Mol Cell Biol. 2014;15(8):509‐524. 25027649 10.1038/nrm3838

[8] Choi GH , Ko KH , Kim JO , et al. Association of miR‐34a, miR‐130a, miR‐150 and miR‐155 polymorphisms with the risk of ischemic stroke. Int J Mol Med. 2016;38(1):345‐356. 27246008 10.3892/ijmm.2016.2609

[9] Chi W , Meng F , Li Y , et al. Downregulation of miRNA‐134 protects neural cells against ischemic injury in N2A cells and mouse brain with ischemic stroke by targeting HSPA12B. Neuroscience. 2014;277:111‐122. 25003713 10.1016/j.neuroscience.2014.06.062

[10] Zheng T , et al. MiR‐130a exerts neuroprotective effects against ischemic stroke through PTEN/PI3K/AKT pathway. Biomed Pharmacother. 2019;117:109117. 31226635 10.1016/j.biopha.2019.109117

[11] Wang Y , Wang M‐D , Xia Y‐P , et al. MicroRNA‐130a regulates cerebral ischemia‐induced blood‐brain barrier permeability by targeting Homeobox A5. FASEB J. 2018;32(2):935‐944. 29070584 10.1096/fj.201700139RRR

[12] Dasari VR , Velpula KK , Kaur K , et al. Cord blood stem cell‐mediated induction of apoptosis in glioma downregulates X‐linked inhibitor of apoptosis protein (XIAP). PLoS One. 2010;5(7):e11813. 20676365 10.1371/journal.pone.0011813 PMC2911373

[13] Rehm M , Huber HJ , Dussmann H , et al. Systems analysis of effector caspase activation and its control by X‐linked inhibitor of apoptosis protein. EMBO J. 2006;25(18):4338‐4349. 16932741 10.1038/sj.emboj.7601295 PMC1570423

[14] Obexer P , Ausserlechner MJ . X‐linked inhibitor of apoptosis protein ‐ a critical death resistance regulator and therapeutic target for personalized cancer therapy. Front Oncol. 2014;4:197. 25120954 10.3389/fonc.2014.00197 PMC4112792

[15] Siegel C , Li J , Liu F , et al. miR‐23a regulation of X‐linked inhibitor of apoptosis (XIAP) contributes to sex differences in the response to cerebral ischemia. Proc Natl Acad Sci U S A. 2011;108(28):11662‐11667. 21709246 10.1073/pnas.1102635108 PMC3136267

[16] Zhang X , Huang L , Zhao Y , et al. Downregulation of miR‐130a contributes to cisplatin resistance in ovarian cancer cells by targeting X‐linked inhibitor of apoptosis (XIAP) directly. Acta Biochim Biophys Sin (Shanghai). 2013;45(12):995‐1001. 24145606 10.1093/abbs/gmt113

[17] Lee S , Hong Y , Won J , et al. Middle cerebral artery occlusion methods in rat versus mouse models of transient focal cerebral ischemic stroke. Neural Regen Res. 2014;9(7):757‐758. 25206884 10.4103/1673-5374.131582 PMC4146278

[18] Chen H , Lin W , Lin P , et al. IL‐10 produces a dual effect on OGD‐induced neuronal apoptosis of cultured cortical neurons via the NF‐kappaB pathway. Aging (Albany NY). 2019;11(23):10796‐10813. 31801113 10.18632/aging.102411 PMC6932931

[19] Longa EZ , Weinstein PR , Carlson S , et al. Reversible middle cerebral artery occlusion without craniectomy in rats. Stroke. 1989;20(1):84‐91. 2643202 10.1161/01.str.20.1.84

[20] Liu DZ , Jickling GC , Ander BP , et al. Elevating microRNA‐122 in blood improves outcomes after temporary middle cerebral artery occlusion in rats. J Cereb Blood Flow Metab. 2016;36(8):1374‐1383. 26661204 10.1177/0271678X15610786 PMC4976655

[21] Yin W , Badr AE , Mychaskiw G , et al. Down regulation of COX‐2 is involved in hyperbaric oxygen treatment in a rat transient focal cerebral ischemia model. Brain Res. 2002;926(1–2):165‐171. 11814419 10.1016/s0006-8993(01)03304-2

[22] He Q‐W , Xia Y‐P , Chen S‐C , et al. Astrocyte‐derived sonic hedgehog contributes to angiogenesis in brain microvascular endothelial cells via RhoA/ROCK pathway after oxygen‐glucose deprivation. Mol Neurobiol. 2013;47(3):976‐987. 23325464 10.1007/s12035-013-8396-8

[23] Ruan L , Wang B , ZhuGe Q , et al. Coupling of neurogenesis and angiogenesis after ischemic stroke. Brain Res. 2015;1623:166‐173. 25736182 10.1016/j.brainres.2015.02.042 PMC4552615

[24] Randolph SA . Ischemic stroke. Workplace Health Saf. 2016;64(9):444. 27621261 10.1177/2165079916665400

[25] Jian R , Yang M , Xu F . Lentiviral‐mediated silencing of mast cell‐expressed membrane protein 1 promotes angiogenesis of rats with cerebral ischemic stroke. J Cell Biochem. 2019;120(10):16786‐16797. 31104315 10.1002/jcb.28937

[26] Sun Y , Gui H , Li QI , et al. MicroRNA‐124 protects neurons against apoptosis in cerebral ischemic stroke. CNS Neurosci Ther. 2013;19(10):813‐819. 23826665 10.1111/cns.12142 PMC6493643

[27] Wang M‐D , Wang Y , Xia Y‐P , et al. High serum MiR‐130a levels are associated with severe perihematomal edema and predict adverse outcome in acute ICH. Mol Neurobiol. 2016;53(2):1310‐1321. 25631713 10.1007/s12035-015-9099-0

[28] Wang W , Wang QI , Yu W , et al. Efficacy of phosphocreatine pre‐administration on XIAP and Smac in ischemic penumbra of rats with focal cerebral ischemia reperfusion injury. Acta Cir Bras. 2018;33(2):117‐124. 29513810 10.1590/s0102-865020180020000003

[29] Mitchelmore C , Gede L . Brain Derived Neurotrophic Factor: epigenetic regulation in psychiatric disorders. Brain Res. 2014;1586:162‐172. 25223903 10.1016/j.brainres.2014.06.037

[30] Liu L , Sun T , Xin F , et al. Nerve growth factor protects against alcohol‐induced neurotoxicity in PC12 cells via PI3K/Akt/mTOR pathway. Alcohol Alcohol. 2017;52(1):12‐18. 27760741 10.1093/alcalc/agw077

[31] Yang H , Zhang H , Ge S , et al. Exosome‐derived miR‐130a activates angiogenesis in gastric cancer by targeting C‐MYB in vascular endothelial cells. Mol Ther. 2018;26(10):2466‐2475. 30120059 10.1016/j.ymthe.2018.07.023 PMC6171076

[32] Liu LU , Liu Y , Feng C , et al. Lithium‐containing biomaterials stimulate bone marrow stromal cell‐derived exosomal miR‐130a secretion to promote angiogenesis. Biomaterials. 2019;192:523‐536. 30529871 10.1016/j.biomaterials.2018.11.007

[33] Lin Z , He H , Wang M , et al. MicroRNA‐130a controls bone marrow mesenchymal stem cell differentiation towards the osteoblastic and adipogenic fate. Cell Prolif. 2019;52(6):e12688. 31557368 10.1111/cpr.12688 PMC6869834

[34] Li Y , Du Y , Cao J , et al. MiR‐130a inhibition protects rat cardiac myocytes from hypoxia‐triggered apoptosis by targeting Smad4. Kardiol Pol. 2018;76(6):993‐1001. 29399759 10.5603/KP.a2018.0040

[35] Wang Y , Gu J , Hu L , et al. miR‐130a alleviates neuronal apoptosis and changes in expression of Bcl‐2/Bax and caspase‐3 in cerebral infarction rats through PTEN/PI3K/Akt signaling pathway. Exp Ther Med. 2020;19(3):2119‐2126. 32104274 10.3892/etm.2020.8415 PMC7027342

[36] Scott FL , Denault J‐B , Riedl SJ , et al. XIAP inhibits caspase‐3 and ‐7 using two binding sites: evolutionarily conserved mechanism of IAPs. EMBO J. 2005;24(3):645‐655. 15650747 10.1038/sj.emboj.7600544 PMC548652

[37] Kairisalo M , Korhonen L , Sepp M , et al. NF‐kappaB‐dependent regulation of brain‐derived neurotrophic factor in hippocampal neurons by X‐linked inhibitor of apoptosis protein. Eur J Neurosci. 2009;30(6):958‐966. 19735291 10.1111/j.1460-9568.2009.06898.x

